# Use of Clinical Outcome Assessments in New Drug Approvals by the US Food and Drug Administration Divisions of Neurology I and II

**DOI:** 10.1001/jamanetworkopen.2022.30530

**Published:** 2022-09-07

**Authors:** Yvette Schein, Holly Fernandez Lynch

**Affiliations:** 1 Perelman School of Medicine, University of Pennsylvania, Philadelphia; 2 Wilmer Eye Institute, Johns Hopkins University School of Medicine, Baltimore, Maryland; 3 Carey Law School, University of Pennsylvania, Philadelphia; 4 Leonard Davis Institute of Health Economics, University of Pennsylvania, Philadelphia

## Abstract

This cross-sectional study examines drug approvals by the US Food and Drug Administration (FDA) Divisions of Neurology I and II to understand the role of clinical outcome assessments in regulatory decisions, including whether and how potential shortcomings are addressed.

## Introduction

The US Food and Drug Administration (FDA) Division of Clinical Outcome Assessments (DCOA) advances the goal of patient-focused drug development “through COA endpoints that are meaningful to patients, valid, reliable and responsive to treatment.”^1^ In contrast to laboratory tests or images, COAs are performance-based assessments or other outcomes reported by patients, clinicians, and nonclinician observers. Because COAs might “be influenced by human choices, judgment, or motivation,”^2^^(p741)^ best practices must be followed to address subjectivity challenges, including validation in specific diseases and use of clear methods for identifying and calculating relevant changes.^3^ Motivated by these considerations, we examined drug approvals by the FDA Divisions of Neurology I and II (DoNI/II) to understand the role of COAs in regulatory decisions, including whether and how potential shortcomings are addressed.

## Methods

In this cross-sectional study, we examined medical, statistical, and “other” clinical review documents available via the Drugs@FDA database for drugs first approved by DoNI/II between 2017 and 2021 and listed in the 2021 COA Compendium.^4^ Areas of focus included end points in pivotal trials, reviewer discussion of COA utility, reviewer alignment on elements relevant to approval, clinical and statistical significance, and conclusions regarding efficacy and safety. Institutional review board requirements were not applicable as this project did not involve research with human participants. We followed the STROBE reporting guideline.

## Results

The 2021 COA Compendium included 14 eligible drugs supported by 32 pivotal trials. Table 1 describes the characteristics of these drugs. All approved indications were deemed serious or life threatening, and reviewers often noted the presence of unmet clinical need (10 [71%]).

**Table 1.** Drugs Approved by the Divisions of Neurology I and II From 2017 to 2021 and Listed in the Clinical Outcome Assessment Compendium

Drug	Approval year	Unmet need^a^	High degree of flexibility^b^	Prior approvals outside the US	Major safety concern^c^
**Acute and chronic migraine**					
Erenumab	2018	No	No	No	No
Fremanezumab	2018	No	No	No	No
Galcanezumab	2018	No	No	No	No
**Amyotrophic lateral sclerosis**					
Edaravone	2017	Yes	Yes	Yes (different indication)	No
**Childhood seizure disorders: LG and DS**					
Cannabidiol (LG, DS)	2018	Yes	Yes	No	No
Stiripentol (DS)	2018	Yes	No	Yes (same indication)	No
**Chorea of Huntington disease**					
Deutetrabenazine	2017	Yes	No	No	No
**Duchenne muscular dystrophy**					
Deflazacort	2017	Yes	Yes	Yes (same indication)	No
**Lambert-Eaton myasthenic syndrome**					
Amifampridine	2018	Yes	No	Yes (same indication)	No
**Multiple sclerosis**					
Ocrelizumab	2017	Yes	No	No	No
Siponimod	2019	Yes	No	Yes (different indication)	No
**Parkinson disease**					
Safinamide	2017	No	No	Yes (same indication)	No
**Polyneuropathy of hereditary transthyretin-mediated amyloidosis**					
Patisiran	2018	Yes	No	No	No
Inotersen	2018	Yes	Yes	Yes (same indication)	Yes

Abbreviations: DS, Dravet syndrome; LG, Lennox-Gastaut syndrome.

^a^ Defined as reviewer’s explicit statement of unmet need in summary review documents.

^b^ Defined as reviewer’s explicit statement of applying a “high degree of flexibility” in deciding what level of evidence was needed to determine efficacy.

^c^ Defined as requiring a Risk Evaluation and Mitigation Strategy.

Table 2 describes COAs used as primary end points in pivotal studies, all of which were blinded and randomized and included additional COAs as secondary outcomes. Of the pivotal studies using COAs as primary end points (31 [97%]), nearly half (15 [48%]) used occurrence diaries with a yes or no outcome, otherwise COAs were rating scales using questions with answer ranges corresponding to severity. For nearly half of the drugs (6 [43%]), secondary COAs, particularly regarding patient function, were used to support determinations of clinical meaningfulness for primary COAs.

**Table 2.** COA Characteristics in Pivotal Studies of Drugs Approved by the Divisions of Neurology I and II From 2017 to 2021 and Listed in the COA Compendium

COA used as primary end point (No. of pivotal studies)^a^	Primary COA					COA used as secondary end point^e^	DCOA consulted	Reviewer disagreement
	Reported by	Type	Reviewer discussion of clinical meaningfulness^b^	Well accepted^c^	Qualified by FDA^d^			
**Summary**								
13 distinct COAs; 18 total	Patient alone or patient plus clinician or caregiver (50%)	Scale (67%)	Independently meaningful (61%)	Yes (56%)	No (100%)	Yes (100%)	No (83%)	No (83%)
**Erenumab**								
Monthly migraine days (3)	Patient	Occurrence diary	Independently meaningful	Yes	No	Yes	Yes	No
**Fremanezumab**								
Monthly migraine days (2)	Patient	Occurrence diary	Independently meaningful	Yes	No	Yes	Yes	No
**Galcanezumab**								
Monthly migraine days (3)	Patient	Occurrence diary	Independently meaningful	Yes	No	Yes	Yes	No
**Edaravone**								
Amyotrophic Lateral Sclerosis Functional Rating Scale–Revised (2)	Clinician	Scale	Independently meaningful	Yes	No	Yes	No	Yes (not COA related)
**Cannabidiol**								
Seizure occurrence (3)	Patient and caregiver	Occurrence diary	Independently meaningful	Yes	No	Yes	No	No
**Stiripentol**								
Seizure occurrence (2)	Patient and caregiver	Occurrence diary	Independently meaningful	Yes	No	Yes	No	No
**Deutetrabenazine**								
Unified Huntington’s Disease Rating Scale (1)	Clinician	Scale	Meaningful with secondary end point support	Yes	No	Yes	No	No
**Deflazacort**								
Medical Research Council Muscle Strength Assessment (2)	Clinician	Scale	Meaningful with secondary end point support	Not mentioned	No	Yes	No	No
**Amifampridine**								
Quantitative Myasthenia Gravis Score (2)	Patient and clinician	Scale	Meaningful with secondary end point support	Not mentioned	No	Yes	No	No
Subject Global Impression (2)	Patient	Scale	Independently meaningful	Not mentioned	No	Yes	No	No
**Ocrelizumab**								
EDSS–Annualized Relapse Rate (2)	Clinician	Scale	Independently meaningful	Yes	No	Yes	No	Yes (not COA related)
EDSS–Confirmed Disability Progression (1)	Clinician	Scale	Independently meaningful	Not mentioned	No	Yes	No	Yes (not COA related)
**Siponimod**								
EDSS–Confirmed Disability Progression (1)	Clinician	Scale	Independently meaningful	Not mentioned	No	Yes	No	No
**Safinamide**								
Unified Parkinson’s Disease Rating Scale (3)	Clinician	Scale	Independently meaningful	Yes	No	Yes	No	No
On time without troublesome dyskinesia (2)	Patient	Occurrence diary	Meaningful with secondary end point support	Yes	No	Yes	No	No
**Patisiran**								
mNIS +7 (1)	Clinician	Scale	Meaningful with secondary end point support	Not mentioned	No	Yes	No	No
**Inotersen**								
mNIS +7 (1)	Clinician	Scale	Meaningful with secondary end point support	Not mentioned	No	Yes	No	No
Norfolk Quality of Life–Diabetic Neuropathy Scale (1)	Patient	Scale	Meaningful with secondary end point support	Not mentioned	No	Yes	No	No

Abbreviations: COA, Clinical Outcome Assessment; DCOA, Division of Clinical Outcome Assessments; EDSS, Expanded Disability Status Scale; FDA, The US Food and Drug Administration; mNIS +7, Modified Neuropathy Impairment Scale +7.

^a^ Number of pivotal studies does not total 32 because some studies used the same COA, 1 study did not use a COA as a primary end point (siponimod), and some studies had coprimary COA end points.

^b^ Indicates that the primary end point may not have been deemed independently clinically meaningful but was considered clinically meaningful when considered in combination with the results of secondary end points.

^c^ Indicates that the COA was used to support previous approvals or described by reviewers as a standard end point for these types of trials.

^d^ Indicates whether the COA has been qualified through the FDA Clinical Outcome Assessment Qualification Program (the FDA does not require a COA to be qualified to support drug approval).

^e^ Secondary COA could be a composite of the primary COA end point or a completely different COA measuring a different aspect of patient experience.

Reviewers rarely commented on COA subjectivity concerns; when they did, it was related to unblinding risk from adverse events. Observed disagreement among reviewers was infrequent and unrelated to COAs. Documented DCOA consultations occurred for only 3 drugs; other discussions of end points likely occurred well before considering a product for approval.

## Discussion

The FDA DoNI/II have accepted COAs as primary and secondary end points to support drug approval. In contrast to occurrence diaries (eg, migraine or seizure rates), scales that require performance and nuanced observation (eg, whether stair climbing is slow or unsteady) may be influenced by factors such as motivation, background conditions, and perspective.^2^ We found little discussion of this bias potential in review documents, but uniform blinding and randomization may reduce the likelihood of bias affecting results. In addition, reviewers sought to weigh multiple aspects of patient experience data in making efficacy judgments, in line with best practices that suggest this triangulation.^3,5,6^ Because review documents demonstrate deference to COAs used in prior approvals, we recommend that the DCOA consider reassessing historically accepted COAs that may not have been subject to stringent review under current standards.

Because analysis was restricted to approved neurology drugs identified as using COAs (ie, listed in the 2021 COA Compendium), further study is needed to assess frequency of approval based on COAs vs other end points, as well as the use of COAs for drugs that are not approved. In addition, this was a small sample of drugs with substantial heterogeneity, limiting generalizability.

As the FDA increasingly incorporates patient experience data into approval decisions, efforts to maintain safeguards including blinding and randomization, reliance on multiple complementary COAs, and collaboration with the DCOA to reassess existing COAs and evaluate new ones will help build confidence in the utility and rigor of these measures.
